# Parents’ perception of child and adolescent mental health problems and their choice of treatment option in southwest Ethiopia

**DOI:** 10.1186/s13034-015-0072-5

**Published:** 2015-08-22

**Authors:** Mubarek Abera, Jeffrey M. Robbins, Markos Tesfaye

**Affiliations:** Department of Psychiatry, College of Health Sciences, Jimma University, Jimma, Ethiopia; Division of Cognitive and Behavioral Neurology, Department of Neurology, Brigham and Women’s Hospital, Harvard Medical School, Boston, USA

**Keywords:** Perception, ‘Child, mental health’, Treatment seeking, Parental attitude, Ethiopia, Traditional beliefs

## Abstract

**Background:**

Parents’ perception and awareness about psychiatric illness in children and adolescents is an important determinant of early detection and treatment seeking for the condition. However, there has been limited information about the perception and awareness of parents about these issues as well as their preferred treatment options in Ethiopia. This study is, therefore, aimed at assessing the perception of parents about psychiatric illness in children and adolescents and their preferred treatment options in Jimma, Ethiopia.

**Method:**

A cross-sectional study was conducted among 532 parents in Jimma City, Ethiopia from April to May 2013. Parents from the city were invited to participate in this study to assess their knowledge on causes, and manifestations of psychiatric illness in children and adolescents as well as their preferred treatment options if their children exhibited signs and symptoms of mental illness.

**Results:**

Nearly three quarters of the parents identified genetic factors while approximately 20 % of them mentioned neuro-chemical disturbance as possible causes of their children’s mental health problems. On the other hand, magic, curse, and sin were mentioned as causes of mental health problems by 93.2, 81.8 and 73.9 % of the parents, respectively. Externalizing behavioral symptoms like “stealing from home, school or elsewhere” and internalizing symptoms like “being nervous in new situations and easily loses confidence” were perceived by 60.9 and 38.2 % of the parents, respectively. The majority (92.7 %) of parents agreed that they would seek treatment either from religious or spiritual healers if their children developed mental illness.

**Conclusions:**

The low level of awareness about internalizing symptoms, the widespread traditional explanatory models as well as preference for traditional treatment options might present significant challenges to utilization of child and adolescent mental health services in this population. Public health intervention programs targeting parental attitude regarding the causes and treatment for child and adolescent mental health problems need to be designed and evaluated for their effectiveness in low-income settings. Additionally, including religious and spiritual leaders in the process of educating members of their respective churches and mosques should also be explored.

## Background

Children are dependent on their parents or care givers to recognize psychopathologies, and seek services, for their mental health problems [1]. For children and adolescents, where there is limited access for health services and mental health professionals, parental perceptions of mental health problems in their children plays a key role in determining service use [2–5]. Pavuluri and colleagues [6] constructed a help seeking pathway that comprises three consecutive steps parents must pass through to eventually access help for their children who are exhibiting symptoms of psychiatric illness. The first is that parents must recognize their child`s symptoms, and the second is that parents must consider getting help for these problems. Lastly, parents must cross their perceived barriers to actually seek help, such as financial and time constraints as well as lack of awareness about the existence and location of such treatment options. Predisposing factors like parental age, gender, race and socio-economic status (SES) are found to influence the help sought for behavioral and emotional disorders among children [7].

Generally, the causes of mental illness in the contemporary world are best explained by a bio-psychosocial model (BPS). A BPS model considers mental illness to be the result of the interaction between biological, psychological and social factors [8, 9]. These factors can act as a risk and/or a protective factor in the development of psychological disorders, making it hard to identify a clear and single identifiable cause for most of the illnesses [10]. There are multiple pathways (different developmental processes or risk factors) leading to one disorder or similar outcome (Equifinality), where as in another situation one pathway (risk factors) may lead to multiple outcomes (Multifinality) [11]. These models of causation and pathway for mental illness are unanimously understood by all mental health professionals across the world regardless of their diverse culture and/or belief system. This common understanding leads for similarities in diagnosis, understanding and managing mental health disorders by psychiatrists and mental health professionals. Many parents, however, especially in developing countries, are more likely to endorse either a disease model or a simple causal model, [12] to understand and explain the causes of mental illness in their children [13]. A disease model describes illness as a syndrome that is either present or absent while a simple causal model implies there is one and only one cause of the illness, and in the absence of this specific cause, the illness would not exist [8, 12]. In the extreme case, it is much easier for parents to believe that there is a primary cause for their child’s mental health problem rather than considering the existence of a the BPS model [14–17].

These parental beliefs about the causes of their child’s mental health problem could potentially impact their ability to recognize and detect different psychopathology as a mental health problem, [18] and this in turn could influence their preference for treatment and help seeking behavior [4, 19–21]. Previous studies have found that traditional and cultural beliefs about the cause of mental illness are greatly widespread phenomena in many African countries contributing to traditional explanatory models of mental illness [22–26]. Furthermore, the choice of treatment options also mainly depended on what was believed to be the root cause of the psychopathology [13, 26].

The World Health Organization (WHO) mental health gap action program (mhGAP), which Ethiopia plans to implement as part of a larger scale-up of mental health services, emphasizes the role of parents and/or care-givers in the management of childhood developmental and behavioral disorders [27]. However, there is limited information about the perception and awareness of Ethiopian parents about child and adolescent mental health problems as well as their attitude towards modern psychiatric services. Such data will be crucial in planning and implementing the scale-up of services for child and adolescent mental health conditions. Therefore, this study is aimed at investigating how Ethiopian parents perceive the common manifestations and causes of psychiatric disturbance in their children and adolescents, as well as to explore where parents might seek treatment if their child exhibited symptoms of a developmental or behavioral disorder.

## Methods and study subjects

### Study setting and period

The study was conducted from April to May 2013 within Jimma City (urban setting) which is located 352 kms Southwest of Addis Ababa, the capital city of Ethiopia. According to the 2007 central statistical agency (CSA) report, the total population of Jimma City was estimated to be 120, 960; of which approximately 36.4 % (44,041) were children age less than 18 years of age [28]. The city has 21 sub-districts and two hospitals, four primary health centers, and two maternal and child health (MCH) clinics as public facilities. Child and adolescent psychiatry services are offered at Jimma University Teaching Hospital (JUTH) within the general psychiatry clinic with a focal person for child and adolescent psychiatry. There is no separate inpatient unit, however, for child and/or adolescent psychiatric patients in Jimma city.

### Study design

A community based cross-sectional quantitative study design was implemented.

### Study subjects

The study was conducted among 532 parents recruited from Jimma city who enrolled in a study on emotional and behavioral disorders among primary school children in the city. This research project was designed to determine the magnitude of child and adolescent emotional and behavioral disorders in Jimma city among children of primary school. Parents, who were invited to participate in the study, rated their child for behavioral and emotional problems based on the parent version of Strength and Difficult Questionnaire (SDQ). This research project also attempted to explore the relationship between emotional and behavioral disorder to academic achievement. The parents of the children who took part in this study were invited for a face-to-face interview using a structured survey questionnaire regarding their perception and awareness of the causes and manifestations of childhood mental health problems as well as their preferred treatment options if their children developed mental illness. The primary schools in the city were clustered into two groups; public and private. A sample of children from all schools were selected randomly. The parents of the selected children were then invited to take part in this study.

### Measurements

Background information of the parents was collected using a structured questionnaire with questions on socio-demographic data (age, sex, religion, and marital status), and socio-economic data (educational status, occupational status, and monthly family income). Perception and awareness of parents about the causes of psychiatric disturbance in children and about their preferred treatment options were assessed by a semi-structured questionnaire developed for the purpose of this study. This questionnaire drew on previous research in this area from low and middle-income countries (LMIC) and took into account the literacy rate and the local context of the Ethiopian community (See “Appendix”). Parents were allowed to list what they thought to be the causes of mental illness in children and adolescents. They were also allowed to list as well as endorse more than one etiologic factor and more than one treatment option from the choices given on the questionnaire. A standard checklist adopted from the SDQ [29] measuring parents’ perceptions of common psychopathologies of children and adolescents was used. SDQ is freely available online and permitted by the author for non commercial use. Additionally, questions derived from the symptoms of child and adolescent mental health problems listed on DSM-IV-TR [30] were included in the checklist. The initial English version of the questionnaire was translated to the local language and was translated back to English independently to ensure semantic equivalence.

### Data collection and data quality assurance

The data was collected by trained data collectors who were fluent in the local language. The questionnaire was pre tested on 5 % of the sample size who were not included in the main study to check for understandability and applicability of the instrument to the local language. Data collectors were trained for 2 days and supportive follow up supervision was given throughout the process of the data collection period. A graduate level mental health specialist supervised the data collection process.

### Statistical analyses

The data was cleaned, coded and analyzed using the SPSS version 20.0 for Windows. It was checked for its distribution and outliers before analysis. Multi co-linearity was checked for independent variables and the variance inflation factor (VIF) was found to range between 1.005 and 1.053 for each of the independent variables. VIF conveys the degree to which multicollinearity amongst the predictors degrades the precision of an estimate. If the value of VIF is higher, there is high probability of multicollinearity amongst the predictors in the model. In general, VIF should not be greater than 10. Descriptive analysis, including frequency distribution, cross tabulation and summary measures were computed. Tests of association between predictors and outcome variables were investigated using Chi square test bivariate and multivariate logistic regression analysis. The association between traditional disease explanatory model and preferred treatment option was computed by controlling for potential confounders. *P* value less than 0.05 was considered statistically significant.

### Ethical clearance

Ethical clearance was granted by the ethical review board of the College of Health Science, Jimma University. Written informed consent was obtained from the study parents.

## Results

### Parents’ socio-demographic characteristics

Of the total (550) parents invited to participate in the study, 532 parents completed the interviews giving a response rate of 96.7 %. Twelve parents refused to participate claiming that they did not have enough time for the interview; whereas six of the remaining parents were not able to give information due to having other responsibilities at home. All of the parents were from urban areas. Of those who completed the interviews, 98 (18.4 %) were male, 348 (65.4 %) were married and 141 (26.5 %) were divorced or widowed. As for religious affiliation, 246 (46.2 %) of the parents were Muslim and 235 (44.2 %) were Coptic Christian. Approximately 53 percent of the parents range in age from 25 to 34 years. The mean age (standard deviation) of the parents was 31.9 (6.5) years. In terms of educational status, 150 (28.2 %) were illiterate, while 236 (44.4 %) and 144 (21.4 %) of the parents had primary and secondary levels of education, respectively. Over two-fifths, 219 (41.2 %) of the parents identified themselves as being a housewife by occupation (Table 1).

**Table 1:** Socio-demographic characteristics of study parents Jimma, Ethiopia, 2013 (n = 532)

Socio-demographic characteristics	Classification	Frequency	%
Sex	Male	98	18.4
	Female	434	81.6
Age	15–24	132	24.8
	25–34	286	53.8
	35–44	97	18.2
	45 and above	17	3.2
Religion	Muslim	246	46.2
	Orthodox c	235	44.2
	Other	51	9.6
Marital status	Married	348	65.4
	Single but gave birth	43	8.0
	Divorced and widowed	141	26.5
Educational status	Illiterate	150	28.2
	Primary school (≤grade 8)	236	44.4
	High school	114	21.4
	College above	32	6.0
Occupational status	Farmer	46	8.6
	Employed	104	19.5
	House wife	219	41.2
	Merchant	163	30.6

### Children’s socio-demographic characteristics

Regarding background information about the children, 288 (54.1 %) of the children were male, the age ranges from 6 to 17 years; 114 (21.4 %) of the children were in the age group of 6–10 years, 317 (59.6 %) of them were in the age group of 11–14 years while the rest 101 (19.0 %) belonged to the age group 15–17 years. Regarding the birth order, 166 (31.2 %) of the children were first born, 136 (25.6 %) second birth, 228 (42.9 %) middle (3–7) and 2 (0.4 %) of the children were the last born in the family birth order. Educational level of the children ranged from grade one to eight.

### Parent’s perception of psychiatric disturbance in children

A significant proportion, 41.6 and 28.1 percent, of the parents recognized some of the externalizing and internalizing symptoms, respectively, as being psychopathology in children and adolescents. The majority, 60.9 and 58.8 %, of the parents cited behaviors such as “Steals from home, school or elsewhere” and “Often loses temper”, respectively as symptoms of psychiatric problems in children. Internalizing behavioral symptoms such as being “nervous in new situations, easily loses confidence” and having “many worries or often seems worried” were also perceived by 38.2 and 37.2 % of the parents as being symptoms of psychiatric illness in children (Table 2).

**Table 2:** Understanding and perception of parents about children’s behavioral, emotional and cognitive manifestations as a mental health problems (n = 532)

Children mental health categories (SDQ)	Symptoms of child mental health problems	Freq	%
Emotional problems	Often complains of headaches, stomach-aches or sickness	78	14.7
	Often unhappy, depressed or tearful	196	36.8
	Many fears, easily scared	234	44.0
	Nervous in new situations, easily loses confidence	198	37.2
	Many worries or often seems worried	203	38.2
	Over all emotional problems	909	34.0
Peer-relationship problems	Picked on or bullied by other youth	123	23.1
	Would rather be alone than with other children	133	25.0
	Generally not liked by other children	114	21.4
	Has no at least one good friend	143	26.9
	Gets along better with adults than with other youth	71	13.3
	Over all peer relationship problems	584	22.0
Hyperactivity problems	Thinks things out before acting	165	31.0
	Restless, overactive, cannot stay still for long time	146	27.4
	Constantly fidgeting or squirming	299	56.2
	Poor attention span, not see work through to the end	217	40.8
	Easily distracted, concentration wanders	232	43.6
	Over all Hyperactivity disorders	1059	39.8
Conduct problems	Quarreling and bullying other children	128	24.1
	Often Lies or cheats	199	37.4
	Often loses temper	313	58.8
	Generally not well behaved, usually doesn’t do what adults request	189	35.5
	Steals from home, school or elsewhere	324	60.9
	Over all conduct problems	1153	43.3
Cluster problems	Internalizing problems	1493	28.1
	Externalizing problems	2212	41.6
Pro-social problems	Not considerate of other people’s feelings	89	16.0
	Not kind to younger children	107	20.1
	Not helpful if someone is hurt, upset or feeling ill	102	19.2
	Refuse to shares readily with other youth, for example books, game	67	12.6
	Often not offers to help others (parents, teachers, children)	69	12.9
	Over all pro social problems	434	16.3
Additional psychopathologies	Truancy from school	64	12.0
	Suicidal thought	142	26.7
	Suicidal behavior	167	31.4
	Sleep problem	343	64.5
	False, fixed and unusual belief	263	49.4
	Hallucination	369	69.4

Prosocial behavior problems such as “refuses to share materials like books and games readily with other youth”, and “often does not offer to help others (parents, teachers, and children)” were the items considered by a smaller proportion of parents (12.6 and 12.9 %) as representing psychopathology in children and adolescents, respectively. Psychotic symptoms like having hallucinations and/or delusions were also recognized by 369 (69.4 %) and 263 (49.4 %) of the parents while suicidal thinking and suicidal behavior were recognized by 142 (26.7 %) and 167 (31.4 %) of the parents as representing symptoms of mental health problems (Table 2).

### Perceived causes of mental health problems in children

Regarding the causes and risk factors for mental illness, approximately 401 (73.4 %) cite genetic factor, 106 (19.9 %) cite neuro-chemical disturbances and 104 (19.5 %) of the parents reported the use of psychoactive substances as biological risk factors for mental illness. However, a remarkably large proportion 496 (93.2 %) cite magic, 435 (81.8 %) cite curse and 393 (73.9 %) of parents endorsed sin as supernatural or spiritual causes of mental illness. These beliefs were more common among illiterate and less educated parents as compared to their literate and more educated counterparts. Academic failure and family related psychosocial problems encountered by children and adolescents were also endorsed as being causes for developing mental illness. Most interestingly 283 (53.2 %) of the parents endorsed at least two risk factors from different domains as being causes of having mental illness (Table 3).

**Table 3:** Perception of parents about causes of children’s mental health problem (n = 532)

Reported risk factors	N	(%)
Supernatural (A)		
Evil spirit	496	93.2
Magic	467	87.8
Attack from devil	412	77.4
Due to sins committed	393	73.9
Will of God	368	69.2
Curse	435	81.8
Biological (B)		
Genetic exposure	401	73.4
Use of psychoactive substances	104	19.5
Neurochemical imbalance	106	19.9
Psychosocial (C)		
Family financial crises	169	31.8
Death of loved family members	416	78.2
Academic failure	94	17.7
Conflictual marriage	359	67.5
Family poverty	299	56.2
Family divorce	316	59.4
Physical or sexual abuse	256	48.1
Reported two or more factors mixed from “A”, “B” or “C”	283	53.2

In the bivariate logistic regression model, divorced parents were ten times more likely to endorse supernatural causes compared to married ones (COR = 9.91; CI = 1.33, 74.09) while parents who were never married were less likely, by over 80 %, to endorse supernatural causes of mental illness as compared with those who were married (COR = 0.18; CI = 0.08, 0.40). Similarly, parents who were illiterate and were less educated (less than 9 year of schooling) were ten times more likely to endorse supernatural causes of mental illness (COR = 9.72; CI = 4.44, 21.25) whereas Coptic Christian parents were over five times more likely to cite supernatural or traditional explanatory models of mental illness than those who identified themselves as being Muslim (COR = 5.33; CI = 2.29, 12.39).

When adjusted for age, sex, religion, marital status, educational and occupational status in the multivariate logistic regression, Coptic Christians were nearly four times more likely to endorse supernatural causes as being, one of the traditional explanatory models of mental illness, than did Muslims (AOR = 3.37; CI = 1.35, 8.42). Parents who were illiterate and were less educated (less than 9 years of schooling) were nearly nine times more likely to endorse supernatural causes as compared with their counterparts who were literate and better educated (AOR = 8.82; CI = 3.79, 20.47). Those who were never married were less likely by over 85 % than those who were married (AOR = 0.15; CI = 0.06, 0.38), while those who were divorced and or were widowed were eight times more likely to endorse supernatural causes of mental illness than those who were married (AOR = 7.98; CI = 1.04, 61.27) (Table 4).

**Table 4:** Final model adjusted for potential confounders to identify independent predictors to endorsed supernatural causes of mental illness, Jimma, Ethiopia

Variables	Category	Sig.	Exp(B)	95% CI for EXP(B)	
				Lower	Upper
Religion	Muslim		1		
	Orthodox christian	.009	3.37	1.35	8.42
Education	Better educated		1		
	Less educated	.000	8.82	3.79	20.47
Occupation	Merchants and employed		1		
	Farmers	.444	.73	.33	1.64
Gender	Male		1		
	Female	.837	1.11	.42	2.94
Marital status	Married	.000	1		
	Single	.000	.15	.06	.38
	Divorced and widowed	.046	7.98	1.04	61.27

### Parents’ help seeking behavior when their children exhibited mental health problems

The majority of parents 368 (69.2 %) reported having expectations that modern mental health care for children is only available in big cities like Addis Ababa than in regional towns. On the other hand, 493 (92.7 %) of the parents agreed that either religious and/or spiritual healers are available within their locality if their children developed any kind of psychiatric problem. Holy water, Rukiya (Holy Quran based religious treatment), praying at home and in the church by religious people were the most commonly mentioned religious and spiritual modalities of treatment for psychiatric disturbance in children.

In the bivariate logistic regression, illiterate and less educated (less than 9 years of schooling) parents were nearly six times more likely to prefer traditional treatment options as compared to their better educated counterparts (COR = 5.61; CI = 3.64, 8.64). Housewives and farmers were 1.5 times more likely to prefer traditional treatment options than were merchants and otherwise employed (COR = 1.51; CI = 1.03, 2.29); Never married parents, however, were less likely, by more than 65 %, to choose traditional treatment options than those who were married (COR = 0.34; CI = 0.17, 0.66); while, Coptic Christians were also two times more likely to prefer traditional treatment options than were Muslims (COR = 1.71; CI = 1.14, 2.55). Those who endorsed supernatural causes of mental illness were approximately ten times more likely to prefer traditional treatment options than their counterparts (COR = 10.30; CI = 4.81, 22.05).

In the final model, adjusting for potential confounders, parents who were illiterate and less educated (less than 9 years of schooling) were nearly five times more likely to prefer traditional treatment options than those who were better educated (AOR = 4.48; CI = 2.82, 7.12); and those who endorsed supernatural causes of mental illness were 4.3 times more likely to prefer traditional treatment options than their counterparts (AOR = 4.33; CI = 1.86, 10.09). Parents who had never married were less likely to prefer traditional treatment options by over 60 % than married parents (AOR = 0.39; CI = 0.18, 0.84) (Table 5).

**Table 5:** Final model adjusted for potential confounders to identify independent predictors of preferred traditional treatment options, Jimma, Ethiopia

Variables	Sig.	Exp (B)	95 % CI for EXP(B)	
			Lower	Upper
Religion				
Muslim		1		
Orthodox christian	.647	1.11	.70	1.77
Education				
Better educated		1		
Illiterate or less than 9 years of schooling	.000	4.48	2.82	7.12
Occupation				
Merchant		1		
Farmers	.268	.77	.49	1.22
Gender				
Male		1		
Female	.718	.88	.49	1.62
Marital status				
Married		1		
Single	.016	.39	.18	.84
Other	.930	1.02	.59	1.76
Endorsed supernatural cause of mental illness				
Yes		1		
No	.001	4.33	1.86	10.09

## Discussion

This study found that the majority of parents recognized that genetic factors may increase the risk for the presence of child and adolescent mental health problems while only a fifth of the parents endorsed neurochemical disturbances and use of psychoactive substances as being risk factors. Parents recognized more externalizing behaviors and psychotic symptoms than internalizing symptoms and suicidal thoughts as representing mental health problems. Furthermore, the vast majority of parents indicated that they would seek treatment from a religious or spiritual healer if their child developed mental illness. Parents’ perception and awareness of psychiatric disturbance in children appears to be an important determinant of early detection and treatment seeking for the condition. Lack of this awareness by the parent may contribute to a majority of affected children who persist with problems that by and large will remain undetected and untreated. This trend inevitably leads to the advancement and chronicity of illness which will continue into adulthood.

In this study, there was greater parental awareness of externalizing than internalizing symptoms as evidences for the presence of psychiatric disturbance in children. The low percentage of parents’ report on internalizing than externalizing symptoms is consistent with research from Western and Non-Western settings [31, 32]. Most studies indicate that parents are less reliable informants of children’s internalizing problems than externalizing behaviors [33]. However, a study from Palestine reported that mothers nearly equally perceived all emotional, behavioral and psychotic symptoms as being suggestive of mental health problems in children and adolescents [34]. The low level of recognition of internalizing symptoms of psychiatric illness in children including suicidal ideation calls for further research on the effectiveness of public mental health intervention programs in raising parental awareness.

Our finding that a large proportion of parents who attribute psychiatric illness in children and adolescents to possession by evil spirits and magic is consistent with findings from other African studies [13, 23]. Similarly a study conducted among the general community in Agaro, Ethiopia, poverty, “God’s will”, “evil spirit” and “stress”, were reported as causes of psychiatric illness in adults [35]. This suggests that the explanatory models for the presence of psychiatric illness in both children and adolescents, as well as in adults might be similar in southwest Ethiopia. The proportion of parents a who attribute psychiatric illness in children and adolescents to psychosocial factors, is comparable to a finding from Lebanon where family-related factors were reported to mediate external stressors and child psychopathology [36]. However, findings from western countries have reported that the majority of parents endorsed bio-psychosocial factors as a cause of child mental health problems [9]. It has previously been suggested that traditional and simple ways to explain causation of disease, [12] are mostly widespread and strongly held beliefs among less developed communities where there is limited education [13, 25].

More interesting in this study is the finding that nearly half of the parents endorsed more than one possible cause of mental illness such as environmental, genetic or organic related causes, which is also consistent with the Palestine study in which some of the mothers perceived multiple causes of child mental health problems, including family problems, parental psychiatric illness and social adversity [34]. These similarities could be explained by cultural similarities to some extent between the Palestinian and Ethiopian parents about their perceptions of attributions. Endorsing more than one possible risk factor, however, doesn’t mean that this study demonstrates the parents’ understanding of the interaction of biopsychosocial factors as a causal model for the existence of psychiatric illness in children and adolescents. Our findings, however could support the idea of equifinality where a number of factors will lead to the same single end point [11].

The finding that majority of the parents’ preference to seek treatment from religious and spiritual healers if their children develop a psychiatric illness appears to be consistent with the predominant explanatory model among the parents. Furthermore, it presents an important challenge in the utilization of the relevant services being developed in the region [37]. First, parents may remain reluctant to bring their child exhibiting developmental or behavioral symptoms to primary care services. Secondly, a supernatural explanatory model for a child’s illness could interfere with parents’ motivation to implement behavioral interventions at home. Similar findings have been reported by studies that investigated treatment seeking for adults with mental illness [38, 39]. This, however, is in contrast to the findings from a study in Palestine where the majority of the mothers preferred Western over traditional types of treatment. This discrepancy might be explained by the parents’ educational status and accessibility of services through the school mental health service and community mobilization for child/adolescent mental health as well as delivery of basic child and adolescent mental health care within primary care in the study setting [40, 41].

Seeking help from traditional spiritual healers has been reported among middle east societies where traditional healers still play a significant role [42]. The effect of parental education on treatment seeking behavior from spiritual healers has been reported by a study from the United Arab Emirates where most educated people prefer to seek help from mental health professional in the event of mental illness in the family [43]. A study conducted in Ethiopia also showed that parents from urban areas are more likely to prefer modern treatment modalities for adults with mental illness compared with their rural counterparts [44] The association of treatment seeking with religion and marital status of parents, however, needs further investigation.

This study suffers the following limitations: (1) the study participants are from one city and do not represent the whole of Ethiopia, (2) the findings may have been influenced by social desirability bias as the data was collected through face to face interviews and (3) existing alternative explanatory models may have been overlooked due to the structured data collection format. Nonetheless, the findings provide crucial information on the potential barriers to the utilization of child mental health services. Policy makers and health programmers need to take into consideration the need for interventions directed at raising public awareness on the causes of childhood mental health conditions.

## Conclusions

The results of this study show that a majority of parents tend to recognize genetic factors to be a risk for psychiatric disturbance in children and adolescents while only a fifth of the parents endorsed neurochemical disturbances and use of psychoactive substances as being risk factors. Parents recognized more externalizing behaviors and psychotic symptoms than they did internalizing symptoms and suicidal thoughts as representing mental health problems. Furthermore, the vast majority of parents indicated that they would seek treatment from a religious or spiritual healer if their child developed mental illness. This low level of awareness about internalizing symptoms coupled with widespread traditional explanatory models as well as preference for traditional treatment options might present significant challenges to the utilization of modern child and adolescent mental health services in this population. Public health intervention programs targeting parental attitude regarding the cause and treatment for child and adolescent mental health problems need to be designed and evaluated for their effectiveness in low-income settings. Additionally, religious and spiritual leaders, if properly educated, could conceivably be powerful advocates of traditional treatment options for children and adolescents exhibiting signs and symptoms of psychiatric illness. This idea is consistent with a new approach taken by staff at the JUTH in treating adults with psychiatric illness. Similarly, teachers at the primary and secondary school levels could be better educated in recognizing psychiatric symptomatology in their students, and could therefore be potentially influential in helping to direct the parents of these students to hospitals and/or clinics who provide these services to children and adolescents. This idea is widely subscribed to in the western world where, in the U.S., for example, one would typically find a school “guidance counselor” on staff whose job is to identify those students who are either exhibiting signs of psychiatric disturbance or who are at risk for doing so. Lastly, the types and modalities of treatment available in hospital and clinic settings need to be explored as well as creating opportunities within any given community to provide mental health services to children and adolescents. This general area, the utilization of resources within a community, is in need of further exploration.
